# Cumulative social risk exposure and risk of cancer mortality in adulthood

**DOI:** 10.1186/s12885-015-1997-z

**Published:** 2015-12-16

**Authors:** Rishi Caleyachetty, Parisa Tehranifar, Jeanine M. Genkinger, Justin B. Echouffo-Tcheugui, Peter Muennig

**Affiliations:** 1 Department of Health Policy and Management, Mailman School of Public Health, Columbia University, New York, NY USA; 2 MRC University Unit for Lifelong Health and Ageing, University College London, London, UK; 3 Department of Epidemiology, Columbia University Mailman School of Public Health, New York, NY USA; 4 Hubert Department of Global Health, Rollins School of Public Health, Emory University, Atlanta, GA USA

**Keywords:** Cumulative social risk, Cancer mortality, Social disparities

## Abstract

**Background:**

Adults in the United States (U.S) can be simultaneously exposed to more than one social risk factor over their lifetime. However, cancer epidemiology tends to focus on single social risk factors at a time. We examined the prospective association between cumulative social risk exposure and deaths from cancer in a nationally representative sample of U.S. adults.

**Methods:**

The study included 8745 adults (aged ≥ 40 years) in the NHANES Survey III Mortality Study over a median follow-up of 13.5 years (1988-1994 enrollment dates and 1988 through 2006 for mortality data). Social risk factors (low family income, low education level, minority race, and single-living status) were summed to create a cumulative social risk score (0 to ≥3). We used Cox proportional hazard models to estimate age- and sex-adjusted hazard ratios (HRs) and 95 % confidence intervals (95 % CI) for the association between cumulative social risk with deaths from all-cancers combined, tobacco-related cancers, and screening-detectable cancers.

**Results:**

Deaths from all-cancers combined (*P* for trend = 0.001), tobacco-related cancers (*P* for trend = <0.001), and lung cancer (*P* for trend = 0.01) increased with an increasing number of social risk factors. As compared with adults with no social risk factors, those exposed to ≥3 social risk factors were at increased risk of deaths from all-cancers combined (HR = 1.8, 95 % CI = 1.3-2.4), tobacco-related cancers (HR = 2.6, 95 % CI: 1.6-4.0), and lung cancer (HR = 2.3, 95 % CI = 1.3-4.1).

**Conclusions:**

U.S. adults confronted by higher amounts of cumulative social risk appear to have increased mortality from all-cancers combined, tobacco-related cancers, and lung cancer. An enhanced understanding of the cumulative effect of social risk factors may be important for targeting interventions to address social disparities in cancer mortality.

## Background

In the United States (U.S.) cancer accounts for 23 % of all deaths, and is the leading cause of death in adults aged 40 to 79 years [1]. However, cancer deaths are not uniformly distributed in the U.S. population with social disparities in cancer mortality being documented in the U.S. as far back as the early 20^th^ century [2]. As with studies on social disparities in health [3–5], studies on social disparities and cancer mortality have mainly focused on single indicators of social disadvantage, such as low education [6–8], low-income [6, 9, 10], minority race/ethnicity [8, 10, 11] and social isolation [12, 13].

But what happens when these social risk factors are experienced together? Socially disadvantaged adults may be exposed to multiple social risk factors simultaneously, with exposure occurring across time and domains [14]. Cumulative social risk exposure refers to experiencing more than one social risk factor at a time, and may better represent the complexity of social disadvantage compared to individual social risk factors [14, 15]. Given that these social risk factors can influence multiple health outcomes and operate via multiple and overlapping mechanisms [16], their co-occurrence as captured through an index of cumulative social risk may lead to larger health adversities, including mortality risk. We have previously shown that exposure to an increasing number of social risk factors were associated with a significantly increased risk of cardiovascular disease (CVD) mortality, premature mortality, and all-cause mortality in U.S. adults [17]. Extant research has not examined the cumulative effect of multiple social risk factors on cancer mortality. Using data from National Health and Nutrition Examination Survey (NHANES) III Mortality Study, a large US population-based cohort study, we created a cumulative risk score based on easily-measured social risk factors and examined the association between cumulative social risk and cancer mortality risk for all cancers combined. We also examined cumulative social risk in association to a combination of tobacco-related cancers, and a combination of screening-detectable cancers as mortality from these cancers represent potentially preventable causes of mortality, and prior research has shown that social disparities are larger for cancers for which there are known preventive risk factors, early detection and treatment methods [16, 18]. Because social risk factors may differentially affect mortality of each site-specific cancer, we also examined cumulative social risk in relation to mortality from the four most common cancer sites (excluding non-melanoma skin cancers) of breast, prostate, colon/rectum, and lung including trachea and bronchus [19].

## Methods

### Study design

The NHANES III, conducted during the 1988-1994 period, is a nationally representative cross-sectional study using a stratified, multistage, and probability cluster design [20]. Data was collected by household interview. We limited the analysis to adults aged ≥40 years who had no history of cancer and had follow-up data on vital status. All participants gave written informed consent and the NHANES study protocol was approved by the National Center for Health Statistics Institutional Review Board.

### Social risk factors

#### Singular social risk factors

The four social risk factors of interest were self-reported through the household questionnaire and include low family income, low education level, minority race/ethnic group, and single-living status. Income was assessed using the poverty income ratio, which is the ratio of the midpoint of observed family income category to the official poverty threshold (scaled to family size), published annually by the US Census Bureau (Series P-60). To define low versus high family income, the poverty income ratio was dichotomized into below 1.00 (below the official definition of poverty) and 1.00 or greater (income above the poverty level) respectively. Education level was assessed as the number of years of education attended and completed, which was then dichotomised into low education (<12 years) and high education (≥12 years). Race was defined as non-white status (i.e. Black, Mexican-American, other Hispanic, Other Race) or white status (non-Hispanic White). Single-living status (a proxy for social isolation and low level of social support) [10] was defined as widowed or divorced, separated or never married, or as married or living as married.

#### Cumulative social risk score

Exposure to each of the four social risk factors was used to create a cumulative social risk score. For each social risk factor, an individual was assigned a value of 0 if unexposed or 1 if exposed. The cumulative social risk score was created by summing the dichotomous variables for each individual social risk factor with a possible score ranging from 0 to 4. Due to the small number of participants with 4 social risk factors, we grouped these individuals with those who had 3 social risk factors.

### Cancer mortality

The National Death Index (NDI) is a central computerized database of all certified death in the United States since 1979. Vital status and cause of death during the follow-up period from baseline in 1988-1994 to December 31, 2006 was conducted by probabilistic matching of NHANES III with the NDI death certificate records. Probabilistic matching was based on several identifiers including social security number, name, birth date, sex, race, state of residence and birth, and marital status [21]. Mortality data was classified according to the International Classification of Diseases, 10th Revision (ICD-10). The ICD-10 codes used for underlying causes of death were C00–C97 for all cancers, C34 for cancer of the lung and bronchus, C18–C20 and C26.0 for colon and rectal cancer, C61 for prostate cancer, and C50 for female breast cancer. Primary outcomes were all-cancers combined, a combination of seven tobacco-related cancers (trachea, bronchus and lung, lip, oral cavity and pharynx, larynx, esophagus, pancreas, bladder, and kidney and renal pelvis), a combination of screening-detectable cancers (breast, cervix uteri, prostate, and colon/ rectum) [22] and all-cause mortality. Secondary outcomes included the four most common sites of cancer deaths of the lung (including trachea and bronchus), breast, prostate and colon/rectum.

### Statistical methods

Of the 10,043 participants aged 40 years or above with no history of cancer at baseline, we excluded those with missing data on the underlying cause of death (n = 62), final mortality status (n = 13), and those with a follow-up of less than one month (n *=* 9). We further excluded participants with missing data on the poverty index ratio (n = 1129), educational level (n = 49) and single-living status (n = 8); no participants had missing race/ethnicity data. The analytic sample was based on the remaining 8745 participants (87 % of the total eligible population). Those excluded due to missing information were less likely to be male (40.4 % vs. 47.6 %; *p* < 0.0003), older (61.3 years vs. 56.1 years; *p* < 0.001), and more likely to have a low education level (43.1 % vs. 29.2 %; *p* < 0.001), belong to a minority race/ethnic group (29.9 % vs. 20.1 %; *p* < 0.001), live as a single person (43.2 % vs. 30.1 %; *p* < 0.001) and have increased mortality (38.4 % vs. 27.5 %; *p* < 0.001).

Baseline characteristics of included participants were calculated. Tetrachoric correlations between each social risk factor were computed. Cox proportional hazards regression models adjusted for age and sex were used to estimate hazard ratios (HR) and 95 % confidence intervals (CI) for the prospective association between single social risk factors and cumulative social risk score (≥3, 2, and 1 vs. 0 social risk factors) respectively, with deaths from all-cancers, tobacco-related cancers, screening-detectable cancers and site-specific cancers. The proportional hazards assumption for the Cox regression model was tested by visually examining –log-log plots of survival versus time and Schoenfeld residuals respectively, and was found not be violated (*p* > 0.05). To test for a linear trend between the number of social risk factors and cancer mortality, a continuous variable for cumulative social risk was included in the model; the statistical significance of the coefficient for that variable was evaluated using the Wald Test [23]. We also tested if the cumulative social risk score was associated with cancer mortality independent of each individual social risk factor. To do so, we conducted analyses that included a variable for cumulative social risk score and each individual social risk factor. If the cumulative effects of social risk factors are significant (*p* > 0.05) when we control for each one of the social risk factors (constituting the cumulative risk score) it suggests that the cumulative social risk score has a greater effect than an individual social risk factor. We examined if the risk of cancer mortality and each social risk factor was modified by sex by adding a cross-product term between each factor and sex to the model and evaluating the coefficient using a Wald Test. All analyses were conducted using Stata version 11.2 (College Station, TX: StataCorp LP) accounting for complex sampling design of NHANES. Weights were applied to all analyses to generate US population estimates.

## Results

The median duration of follow-up was 13.5 (range: 0.1-18.2) years. From 1988 to 2006, there were 3489 deaths (1813 in males and 1676 in females). Of these deaths, 699 deaths were from all-cancers combined (419 in males and 280 in females) 308 were from tobacco-related cancers (203 in males and 105 in females) and 166 from screening-detectable cancers (101 in males and 65 in females). The characteristics of participants are shown in Table 1. Compared to males, females were more likely to have low family income (8.7 % vs. 12.4 %; *p* < 0.001), belong to a minority race/ethnic group (18.9 % vs. 21.2 %; *p* = 0.009), live as a single person (21.4 % vs. 38 %; *p* = <0.001) and be exposed to ≥ 3 social risk factors (6.5 % vs. 10.0 %; *p* < 0.001). There was no evidence for interactions between each social risk factor and sex (p-value, test for interaction > 0.05) hence analyses were not stratified. Tetrachoric correlations between individual social risk factors were all positive and statistically significant (Table 2).

**Table 1:** Characteristics among 8745 adults aged 40 years and over, NHANES III Mortality Follow-up study: 1988–1994 to 2006

	Male (n = 4184)	Female (n = 4561)	*p*-value
Age, mean years	55.2	57.0	<0.001
Social risk factors, n (%)			
Low family income	768 (8.7)	1068 (12.4)	<0.000
Low educational level	2046 (29.8)	2097 (28.7)	0.473
Minority race/ethnic group	2182 (18.9)	2304 (21.2)	0.009
Single-living	1009 (21.4)	2185 (38.0)	<0.001
Number of social risk factors, n (%)			
0	1015 (48.1)	892 (39.7)	<0.001
1	1295 (32.9)	1284 (33.2)	
2	1075 (12.5)	1201 (17.2)	
≥ 3	799 (6.5)	1184 (10.0)	
Cancer mortality			
All-site, n (%)	419 (7.4)	280 (5.5)	0.004
Tobacco-related cancer, n (%)	203 (4.0)	105 (2.2)	0.473
Screening-detectable cancer, n (%)	101 (1.4)	65 (1.3)	0.732
Site-specific cancer mortality			
Lung Cancer, n (%)	143 (3.1)	76 (1.8)	0.009
Colorectal Cancer, n (%)	45 (0.7)	22 (0.6)	0.650
Prostate Cancer, n (%)	55 (0.7)	-	NA
Female Breast Cancer, n (%)	-	36 (0.6)	NA

*NA* not applicable

Low family income was defined as the poverty income ratio < 1.00 (below the official definition of poverty)

Low education level was defined as < 12 years (< high school)

Minority racial/ethnic group was defined as adults who were Black, Mexican-American, other Hispanic and Other

Single-living status (proxy for social isolation and low level of social support) was defined as persons widowed, divorced, separated or never married

**Table 2:** Tetrachoric correlations of social risk factors

	Low family income	Low education level	Minority race/ethnic group	Single-living
Low family income	1.0			
Low education level	0.5*	1.0		
Minority race/ethnic group	0.5*	0.4*	1.0	
Single-living	0.3*	0.1*	0.1*	1.0

*p < 0.05

Table 3 shows the association between the singular social risk factors and death from all-cancers combined, tobacco-related cancers, screening-detectable cancers and site-specific cancers, adjusted for age and sex. Adults with low family income had an increased risk of death from all-cancers combined (HR = 2.1, 95 % CI: 1.6-2.8), tobacco-related cancers (HR = 2.8, 95 % CI: 2.0-4.1), and lung cancer (HR = 2.7, 95 % CI: 1.7-4.3). Adults with low educational level had an increased risk of death from screening-detectable cancers (HR = 2.0 95 % CI: 1.1-3.7), and female breast cancer (HR = 3.0, 95 % CI: 1.3-6.9). Adults belonging to a minority race/ethnic group had an increased risk of death from all-cancers combined (HR = 1.3, 95 % CI: 1.0-1.5), tobacco-related cancers (HR = 1.5, 95 % CI: 1.0-2.1), and screening-detectable cancers (HR = 1.8, 95 % CI = 1.0-1.1). Adults living as single people did not have an increased risk of death from cancer.

**Table 3:** Association between single social risk factors and deaths from all-cancers combined, tobacco-related cancers, screening-detectable cancers, all-causes, and site-specific cancers, NHANES III Mortality Follow-up study: 1988–1994 to 2006

	All-cancers combined				Tobacco-related cancers				Screening-detectable cancers			
	N	Rate/1000 y (95 % CI)		HR^a^ (95 % CI)	N	Rate/1000 y (95 % CI)	HR^a^ (95 % CI)		N	Rate/1000 y (95 % CI)	HR^a^ (95 % CI)	
Low family income	188	9.0 (7.7-10.3)		2.1 (1.6-2.8)	95	4.5 (3.7-5.5)	2.8 (2.0-4.1)		41	2.0 (1.4-2.7)	1.3 (0.6-2.6)	
Low educational level	390	8.3 (7.5-9.1)		1.2 (0.9-1.5)	165	3.5 (3.0-4.1)	1.2 (0.8-1.6)		105	2.2 (1.8-2.7)	2.0 (1.1-3.7)	
Minority racial/ethnic group	357	6.3 (5.7-7.0)		1.3 (1.0-1.5)	162	2.9 (2.4-3.3)	1.5 (1.0-2.1)		96	1.7 (1.4-2.1)	1.8(1.0-1.1)	
Single-living	263	7.5 (6.6-8.4)		1.0 (0.8-1.3)	124	3.5 (2.9-4.2)	1.3 (0.9-1.8)		58	1.6 (1.2-2.1)	0.7 (0.5-1.0)	
	Lung Cancer			Colorectal Cancer			Prostate Cancer			Female Breast Cancer		
	N	Rate/1000 y	HR^a^	N	Rate/1000 y	HR^a^	N	Rate/1000 y	HR^b^	N	Rate/1000 y	HR^b^
		(95 % CI)	(95 % CI)		(95 % CI)	(95 % CI)		(95 % CI)	(95 % CI)		(95 % CI)	(95 % CI)
Low family income	66	3.1 (2.4-4.0)	2.7 (1.7-4.3)	10	0.5 (0.2-0.9)	0.8 (0.2-3.2)	14	0.7 (0.4-1.1)	1.2 (0.5-3.0)	12	0.7 (0.3-0.1)	1.1 (0.6-2.3)
Low educational level	119	2.5 (2.1-3.0)	1.3 (0.9-1.9)	34	0.7 (0.5-1.0)	1.3 (0.5-3.3)	43	0.9 (0.7-1.2)	2.5 (0.9-7.0)	21	0.5 (0.3-0.8)	3.0 (1.3-6.9)
Minority racial/ethnic group	109	1.9 (1.6-2.3)	1.3 (0.8-2.1)	34	0.6 (0.4-0.8)	1.4 (0.7-2.7)	32	0.6 0.4-0.8	1.7 (0.8-3.5)	25	0.5 (0.3-0.7)	2.5 (0.8-8.2)
Single-living	81	2.3 (1.8-2.9)	1.1 (0.7-1.6)	22	0.6 (0.4-0.9)	0.7 (0.3-1.3)	13	0.4 (0.2-0.6)	0.9 (0.3-2.9)	19	0.6 (0.4-0.9)	0.8 (0.4-1.8)

*HR* Hazard Ratio, *95 % CI* 95 % Confidence Intervals

^a^Adjusted for age and sex

^b^Adjusted for age

Table 4 shows the association between the cumulative social risk and death from all-cancers combined, tobacco-related cancers, screening-detectable cancers and cause-specific cancers. Adults exposed to 3 or more social risk factors were at greater risk of mortality from all-cancers combined (HR = 1.8, 95 % CI: 1.3-2.4), tobacco-related cancers (HR = 2.6, 95 % CI: 1.6-4.0) and lung cancer (HR = 2.3, 95 % CI: 1.3-4.1) compared to adults exposed to no social risk factors. Hazard ratios for deaths from all-cancers combined, tobacco-related cancers, lung cancer significantly increased with an increasing number of social risk factors (all-cancers combined: *p* for trend = 0.001; tobacco-related cancers: *p* for trend = <0.001; lung cancer: *p* for trend = 0.01).

**Table 4:** Association between the number of social risk factors and deaths from all-cancers combined, tobacco-related cancers, screening-detectable cancers, all-causes, and site-specific cancers, NHANES III Mortality Follow-up study: 1988–1994 to 2006

	All-cancers combined				Tobacco-related cancers				Screening-detectable cancers			
	N	Crude Rate/1000 y		HR^a^	N	Crude Rate/1000 y		HR^a^	N	Crude Rate/1000 y		HR^a^
		(95 % CI)		(95 % CI)		(95 % CI)		(95 % CI)		(95 % CI)		(95 % CI)
0	127	5.1 (4.2-6.0)		1.0 (ref)	58	2.3 (1.8-3.0)		1.0 (ref)	24	1.0 (0.6-1.4)		1.0 (ref)
1	200	6.3 (5.4-7.2)		1.1 (0.8-1.5)	80	2.5 (2.0-3.1)		1.0 (0.6-1.6)	49	1.5 (1.1-2.0)		1.6 (0.8-3.2)
2	179	6.6 (5.7-7.7)		1.3 (1.0-1.8)	74	2.7 (2.1-3.4)		1.5 (1.1-2.2)	48	1.8 (1.3-2.3)		1.9 (0.8-4.3)
≥3	193	8.6 (7.4-9.9)		1.8 (1.3-2.4)	96	4.3 (3.5-5.2)		2.6 (1.6-4.0)	45	2.0 (1.5-2.7)		1.8 (0.8-4.1)
P_trend_	0.001				<0.001				0.095			
	Lung cancer			Colorectal cancer			Prostate cancer			Female Breast cancer		
	N	Crude Rate/1000 y	HR^a^	N	Crude Rate/1000 y	HR^a^	N	Crude Rate/1000 y	HR^b^	N	Crude Rate/1000 y	HR^b^
		(95 % CI)	(95 % CI)		(95 % CI)	(95 % CI)		(95 % CI)	(95 % CI)		(95 % CI)	(95 %CI)
0	47	1.9 (1.4-2.5)	1.0 (ref)	13	0.5 (0.3-0.9)	1.0 (ref)	7	0.3 (0.1-0.6)	1.0 (ref)	4	0.2 (0.0-0.5)	1.0 (ref)
1	55	1.7 (1.3-2.2)	1.0 (0.0.6-1.6)	26	0.8 (0.5-1.1)	1.3 (0.5-3.7)	12	0.4 (0.2-0.7)	1.9 (0.5-7.7)	9	0.3 (0.1-0.6)	1.8 (0.4-7.0)
2	53	2.0 (1.5-2.6)	1.4 (0.9-2.4)	14	0.5 (0.3-0.9)	0.8 (0.3-2.6)	24	0.9 (0.6-1.3)	3.1 (0.9-10.4)	9	0.4 (0.2-0.7)	2.6 (0.5-13.5)
≥3	64	2.9 (2.2-3.6)	2.3 (1.3-4.1)	14	0.6 (0.3-1.0)	1.2 (0.3-6.0)	12	0.5 (0.3-0.9)	2.0 (0.5-7.1)	14	0.7 (0.4-1.2)	2.4 (0.8-7.6)
P_trend_	0.012			0.955			0.586			0.047		

*HR* Hazard Ratio, *95 % CI* 95 % Confidence Intervals

^a^Adjusted for age and sex

^b^Adjusted for age

The cumulative effects of social risk factors on death from all-cancers combined, tobacco-related cancers, and lung cancer became non-significant when we controlled for low family income, except for when breast cancer mortality was the outcome (results not shown).

## Discussion

This study finds an increased risk of deaths from all-cancers combined, tobacco-related cancers, and lung cancer with increasing number social risk factors in a large nationally representative sample of U.S. adults. Adults exposed to 3 or more social risk factors had a 1.8-to 2.6-fold increased risk of all-cancer and tobacco-related cancer mortality.

Similar to much of the history of thinking about social disparities in the U.S. [24], research on social disparities and cancer mortality has typically been framed in terms of single social risk factors [25–28], with a focus on race or education. For example, in a study examining selected cancer mortality rates from 26 U.S. states, educational attainment reported by next of kin was the only indicator of social disadvantage that was used, because this was the only indicator recorded on death certificates [28]. In the U.S. National Longitudinal Mortality Study, the risk of cause-specific cancer mortality was associated with black race [10]. However, as with most such studies, income and education were added as covariates, “isolating” the effect of black race, but providing very little information about the actual risk faced by most black people in the U.S. As we have shown here, social risk factors may co-occur and multiple rather than single social risk factors can have a greater impact on cancer mortality.

We found the cumulative effects of social risk factors on all-cancers combined, tobacco-related cancers, and lung cancer were driven by low family income. Tobacco smoking among low-income adults is known to be more prevalent, but also quit attempts are less likely to be successful [29]. Low-income status can limit monetary resources that can help smokers achieve cessation, limit the person’s capability of utilizing these monetary resources for cessation, as well as influencing the likelihood of predictors of cessation behaviour (e.g. motivational factors, nicotine dependence) [29]. Race/ethnicity and education each contribute to future income level, so this may partly explain why overall and cause-specific cancer mortality was driven to a larger extent by low family income in this middle-to-old aged cohort.

Determining the influence of cumulative social risk on cancer mortality may help inform the design of effective interventions to address social disparities in cancer mortality in the U.S. Recently, the US Preventative Services Task Force (USPSTF) has recommended annual screening for lung cancer with low-dose computed tomography in adults aged 55 to 80 years who have a 30 pack-year smoking history and currently smoke or have quit within the past 15 years [30]. It has been suggested that it might be also important to monitor lung cancer incidence and stage at diagnosis by race so that resources can be put in place to identify groups that may need targeted screening efforts [31, 32]. However, only monitoring race without taking into account financial barriers may not itself be very effective in eliminating the disparity in lung cancer mortality. The reason is that policies primarily targeted to an individual social risk factor may not fundamentally address issues related to other social risk factors among the proportion of socially disadvantaged adults exposed to multiple social risk factors.

A major strength of the study is that we used a cumulative social risk score based on easily-measured social risk factors to prospectively examine the association between cumulative social risk and cancer mortality in a large nationally representative sample of the U.S. population over a sufficiently long period. Furthermore, percentages of death according to site closely reflect published cancer statsitics [33]. However, several limitations of our study merit consideration. First, the small number of deaths limited our power to detect associations between single social risk factor exposures or cumulative exposure to social risk factors and cancer mortality, especially for site-specific cancers. This may also have limited our ability to detect if risk of cancer mortality and each social risk factor was significantly modified by sex. Second, the finding that adults living as single people were not at increased risk of death from cancer, could be because single-living is a poor proxy for social isolation and reduced social support. This may also lead to attenuate any association between exposure to cumulative social risk and cancer mortality. A meta-analytic review examining the extent to which social relationships influence the risk for mortality, found that the association was strongest for more complex measures of social integration [34]. Third, the use of a cumulative social risk score, such as this, acknowledges that social risk factors tend to co-occur and also makes the implicit assumption that each form of social risk factor carries the same level of risk on cancer mortality. Weighting the cumulative social risk score might be useful in this scenario, however there are several important caveats to doing this. Given that proposed weights are based on the relative association of each social risk factor with the outcome, different weights would end up being proposed for different cancer mortality outcomes for the same social risk factors. Also, specific weights for social risk factors would be given based on specific datasets. Thus, it is not certain that weights derived from this dataset would be generalizable to another dataset. Moreover, considering that these social risk exposures occur across the life course, longitudinal analyses from earlier in the life course would be required in order to more fully understand the contemporaneous and cumulative impact of multiple social risk factor exposure that may underlie social disparities in cancer mortality. We also made the assumption that that components of the cumulative social risk metric have no temporal order. Exposure to one social risk factor may lead to another social risk factor and thus tend to co-occur (chains of risk), or social risk factors may follow one another sequentially but risk of mortality is not increased until the effect of the final exposure in the chain (“trigger effect”) [35]. Chronicity of exposure to social risk factors are also ignored in the cumulative social risk score, and may also be important for influencing cancer mortality. Future research might overcome some of the aforementioned concerns, by collecting data on the age of when the adult was exposed to a specific social risk factor. Fourth, we were not able to examine the potential contribution of mediators including cigarette smoking, obesity, poor diet, physical inactivity, health insurance status and occupational exposures either because of the high percentage of missing data on these variables in the dataset or lack of availability. Finally, those with missing information were more likely to belong to a minority race/ethnic group, have a low education level, be living as a single person and have higher mortality. Thus, the current findings are probably underestimates of the true magnitude of the association between cumulative social risk and cancer mortality. We also acknowledge the possibility that residual confounding may attenuate the strong HRs estimates in this study.

## Conclusion

We found that exposure to an increasing number of social risk factors increased the risk of death from all-cancers combined, tobacco-related cancers, and lung cancer in U.S. adults. Future research on the multiple social risk factor exposure earlier in the life course is required in order to better understand the association between cumulative social risk and cancer mortality in the U.S.

## Abbreviations

CVD: Cardiovascular disease
CI: Confidence intervals
HR: Hazard ratio
ICD-10: International Classification of Diseases, 10th Revision
NDI: National Death Index
NHANES: National Health and Nutrition Examination Survey
U.S.: United States
